# Foliar Phenolic Compounds in Norway Spruce with Varying Susceptibility to *Chrysomyxa rhododendri*: Analyses of Seasonal and Infection-Induced Accumulation Patterns

**DOI:** 10.3389/fpls.2017.01173

**Published:** 2017-06-30

**Authors:** Andrea Ganthaler, Wolfgang Stöggl, Ilse Kranner, Stefan Mayr

**Affiliations:** ^1^Faculty of Biology, Department of Botany, Institute of Botany, University of InnsbruckInnsbruck, Austria; ^2^alpS – Centre for Climate Change AdaptationInnsbruck, Austria

**Keywords:** conifers, flavonoids, pathogenic fungus, pathogen resistance, *Picea abies*, stilbenes

## Abstract

Secondary phenolic metabolites are involved in plant responses to various biotic stress factors, and are apparently important for the defense against fungal pathogens. In this study, we investigated their role in defense against the rust *Chrysomyxa rhododendri* in Norway spruce. The fungal pathogen undergoes a seasonal lifecycle with host shift; after overwintering in rhododendron shrubs, it attacks the sprouting current-year spruce needles and causes needle fall in autumn. Repeated infections lead to reduced timber yield and severe problems with rejuvenation in subalpine Norway spruce forests. Trees with varying susceptibility to infection by *C. rhododendri* were selected and foliar phenolic composition was assessed using UHPLC-MS. We report on seasonal accumulation patterns and infection-related changes in the concentrations of 16 metabolites, including flavonoids, stilbenes, simple phenylpropanoids and the precursor shikimic acid, and their correlation with the infection degree of the tree. We found significant variation in the phenolic profiles during needle development: flavonoids were predominant in the first weeks after sprouting, whereas stilbenes, picein and shikimic acid increased during the first year. Following infection, several flavonoids and resveratrol increased up to 1.8 fold in concentration, whereas picein and shikimic acid were reduced by about 70 and 60%, respectively. The constitutive and early stage infection-induced concentrations of kaempferol, quercetin and taxifolin as well as the late stage infection-induced concentrations of stilbenes and picein were negatively correlated with infection degree. We conclude that a combination of constitutive and inducible accumulation of phenolic compounds is associated with the lower susceptibility of individual trees to *C. rhododendri*. The potentially fungicidal flavonoid aglycones may limit hyphal growth and prevent development of infection symptoms, and high levels of stilbenes may impede the infection of older needles. The presented results underline a highly compound-specific seasonal accumulation and defense response of Norway spruce and may facilitate the selection of promising trees for breeding programs.

## Introduction

Conifers synthesize a large range of secondary phenolic metabolites, and some of them accumulate in high concentrations in bark, roots and needles (Erdtman and Harmatha, 1979; Pan and Lundgren, 1995; Slimestad, 2003). These metabolites are involved in plant response to numerous biotic stress factors, including fungal pathogens (Hammerschmidt, 2005; Witzell and Martín, 2008; Chong et al., 2009). Although the underlying mechanisms that confer protection and the localization and compartmentation of most compounds are still unclear, several functions have been proposed. Phenolics can have direct fungicidal or antioxidant properties, or contribute indirectly to resistance by modulating the activity of other phytochemicals, representing precursors of defense-related compounds and polymers, and enhancing mechanical barriers (Schultz and Nicholas, 2000; Treutter, 2006; Cvikrová et al., 2008). Moreover, different phenolic compounds may confer resistance by synergy effects, or challenge the pathogen due to variable concentration levels (Wallis et al., 2008; Edenius et al., 2012).

Phenolic compounds constitutively accumulate in healthy plants, and can be induced in response to infection (Evensen et al., 2000; Danielsson et al., 2011; Hammerbacher et al., 2011). Especially stilbenes, widespread compounds in the Pinaceae family, are frequently induced by pathogen attack (Chong et al., 2009; Jeandet et al., 2010) and varying concentrations in the tissue have been associated with intraspecific variation of host plant susceptibility to infection (Lieutier et al., 2003). Induced phenolics accumulation can be due to a stimulation of the phenylpropanoid pathway and/or rapid translocation and modification of present compounds (Matern and Kneusel, 1988; Cvikrová et al., 2008; Hammerbacher et al., 2011). Most common modifications, altering the hydrophilicity, stability, subcellular localization, and bioactivity of the compounds are methoxylation, oligomerization, glycosylation, and isomerization (Chong et al., 2009).

**Figure 1:**
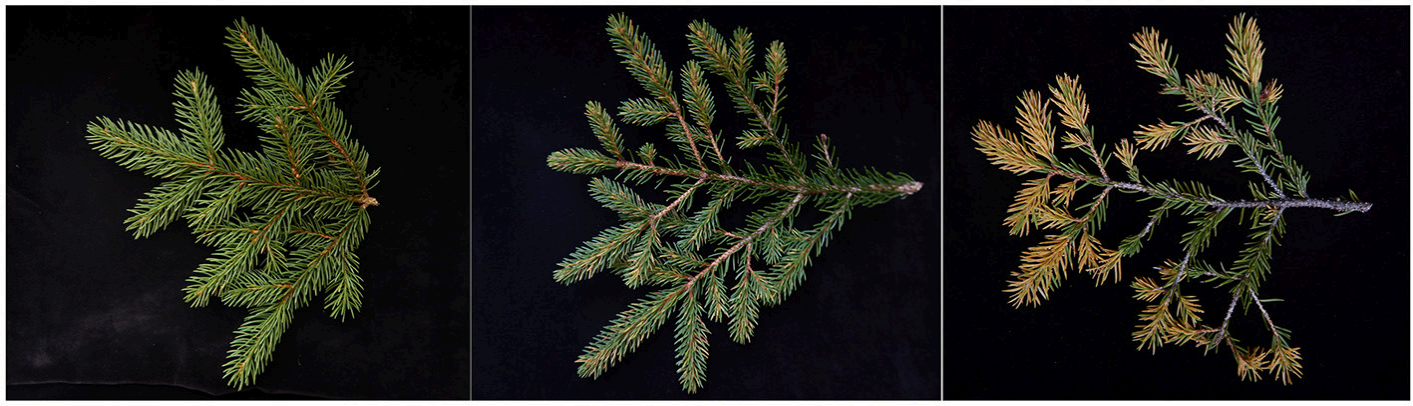
Norway spruce twigs with infection symptoms. Twigs from three trees with varying susceptibility to *C. rhododendri* were harvested in August. Note that only current-year-needles are affected, whereas reduced needle density in previous-year branch sections indicate past infections.

In the last three decades, numerous phenolic compounds were isolated from Norway spruce and their structures identified (Hoque, 1986; Kicinski and Kettrup, 1987; Strack et al., 1989; Pan and Lundgren, 1995; Slimestad and Hostettmann, 1996; Slimestad et al., 1999). Several exhibited *in vitro* toxicity toward fungal cells or were detected in varying concentrations following infection *in vivo* (compare the review of Witzell and Martín, 2008). In addition, constitutive and induced concentrations differed between clones and attempts have been made to use phenolic metabolites as predictors of spruce quantitative resistance to insect and microbial attacks (Lieutier et al., 1997; Brignolas et al., 1998; Danielsson et al., 2011). A connection between phenolics levels and susceptibility to infection was also found for spruce attacked by the needle bladder rust (Ganthaler et al., 2017). This rust species (*Chrysomyxa rhododendri*; De Bary, 1879) is a serious, but underinvestigated pathogen of Norway spruce in the European Alps. It causes distinct needle yellowing during summer followed by needle fall in autumn, and completes the life cycle by overwintering in the leaves of rhododendron plants. Repeated infections lead to reduced timber yield and severe problems with rejuvenation (Oberhuber et al., 1999; Ganthaler et al., 2014). Nevertheless, high variation of susceptibility of spruce to this pathogen was repeatedly reported (Figure 1; Dufrénoy, 1932; Oechslin, 1933; Mayr et al., 2010; Ganthaler et al., 2017) and interestingly, even in years with severe outbreaks, some individual trees can suffer significantly less from fungal infection compared to surrounding trees. Although spruce resistance to *C. rhododendri* is clearly a quantitative trait with large phenotypic variation, trees with remarkable low susceptibility are extremely rare.

The detailed role of phenolics in this varying susceptibility is far from clear, but earlier results on this pathosystem (Ganthaler et al., 2017) and on willow infected by *Melampsora* (Hakulinen et al., 1999; Hjältén et al., 2007) indicated that phenolic secondary compounds can limit the growth of rust fungi immediately after infection and prevent the development of infection symptoms. Furthermore, previous studies underlined the relevance of seasonal variations and infection-induced changes in the accumulation of individual foliar phenolic compounds. This variability is of particular importance for *C. rhododendri* infections, as the pathogen infects only the new sprouting needles in early summer (Ganthaler and Mayr, 2015) and can spread only within a single infected needle. In addition, previous studies indicate different accumulation patterns of flavonoids and stilbenes during needle development in spruce (Strack et al., 1989; Slimestad and Hostettmann, 1996; Slimestad, 1998), but studies on continuous seasonal courses are missing. Enhancing our knowledge of seasonal and infection-induced variation of phenolic profiles could help to better understand the resistance mechanisms of *Picea abies* to *C. rhododendri* and support the development of strategies to handle this detrimental forest pathogen.

Here we report (a) on the accumulation of 16 phenolic compounds during needle development, (b) infection-related changes in needles infected by *C. rhododendri* and (c) correlations of phenolics concentrations before and during symptom development with infection intensity. Analyses were conducted of ten trees with varying infection degree located in three subalpine forest sites. Healthy and infected needles were analyzed separately to identify infection-induced concentration changes on the needle level. The study aimed at understanding the variability of phenolic compounds concentrations in healthy and infected needles of Norway spruce and the role of constitutive and induced phenolic levels for varying susceptibility to infection by *C. rhododendri*.

## Materials and methods

### Rust quantification and sampling of trees

Trees were selected from three different subalpine forest sites in Tyrol, Austria with high *C. rhododendri* infection pressure: Praxmar (1,614 m s. l., N 47° 09.495, E 11° 08.201, *n* = 3), Rinn Fluchtalweg (1,655 m s. l., N 47° 14.090, E 11° 30.916, *n* = 3) and Rinn Kriegerkapelle (1,667 m s. l., N 47° 13.971, E 11° 30.916, *n* = 3). These nine trees showed high but varying infection intensities and were used for the analysis of compounds accumulation during needle development (healthy needles) and changes following infection (infected needles). In addition, one of the extremely rare trees with hardly any susceptibility, hereafter termed “low susceptibility,” which was available for repeated sampling, was analyzed. This resistant tree, “PRA-R,” of the Praxmar site was already used in previous studies (Mayr et al., 2001, 2010). It was included in the correlation analysis between compounds concentrations and infection degree, and its seasonal variations in phenolic concentrations is presented separately in the supplement to highlight the phenolic characteristics of this exceptional example of enhanced resistance.

In 2015, infection degrees for all ten trees were determined by assessing the percentage of current-year needles with infection symptoms, and for the years 2011–2014 by assessing the percentage of needle loss due to infection on sampled twigs. Selected trees were not affected by other foliar pathogens or insects causing needle loss. Missing needles were estimated according to the number of empty needle bases and by using a discrete scale with 10% intervals. Monitoring over several years is important, as the percentage of infected needles is influenced by local spore densities and weather conditions during the infection period (Ganthaler and Mayr, 2015). Consequently, the mean infection degree of several years is expected to represent the general susceptibility of the tree to infection by *C. rhododendri* and was used for correlation analyses with compounds concentrations.

For each tree and sampling date, three branches (about 40 cm long and including several years of growth) were collected at breast height between 10 and 12 a.m. Central European Time and transported in dark plastic bags at ambient temperature and within 2 h to the laboratory. Needles were cut, separated into previous-year, current-year healthy and infected needles (where applicable), and immediately stored in cryo vials at −80°C. Sampling was carried out periodically from bud swelling in early June to early October 2015. To account for the expected rapid changes in sprouting needles, sampling was intensified in the first weeks. Previous-year needles were analyzed on three sampling dates (26 June, 2 July and 27 August), as preliminary analyses showed minimal seasonal variation for older needles (compare also Slimestad, 1998). Infected needles were separated from healthy needles as soon as first infection symptoms appeared and until infected needles were shed (July to August). Additionally, bud sprouting and length of sprouts were monitored.

### Identification and quantification of phenolic compounds

Sample preparation, extraction and analysis of phenolic metabolites were conducted as described in Ganthaler et al. (2017). Briefly, needles were freeze-dried for 72 h and homogenized for 8 min at 2000 rpm in a microdismembrator (Mikro-Dismembrator U, Braun Biotech International, Melsungen, Germany). The powder was quickly transferred into vials and 10 mg was extracted two times for 20 min each at 50°C with 1 ml 95% (v/v) ethanol, containing 2 μmol L^−1^ orientin, pinosylvin and naringin as internal quantification standards. Each extract was diluted 1:2 and 1:50 to obtain a 50:50 ethanol/water (v/v%) solution and both dilutions were analyzed to consider the different concentration ranges of the compounds in spruce needles, and to improve detection of low and high values (quantification thresholds for the individual compounds varied between 0.001 and 0.027 μmol g^−1^). Sixteen phenolic compounds, including stilbenes, flavonoids, simple phenylpropanoids and the precursor shikimic acid (Table 1), were identified and quantified by liquid chromatography-mass spectrometry (UHPLC-MS), using an ekspert ultraLC 100 UHPLC system coupled to a QTRAP 4500 mass spectrometer (both from AB SCIEX, Framingham, MA, USA). Detection and quantification of the single metabolites were conducted using authentic samples of all substances and calibration curves. For compounds separation, a reversed-phase UHPLC column (NUCLEODUR C18 Pyramid, EC 50/2, 50x2 mm, 1.8 μm, Macherey-Nagel, Düren, Germany) with a 4 × 2 mm guard column connected ahead was used. Run time was set to 8 min and mobile phases were 0.1% formic acid (v/v) (A) and acetonitrile (B), starting with 5% B followed by a gradient to 70% B (5 min), rinsing at 100% B (5:01 to 6 min) and equilibration at 5% B (6:30 to 8 min). The injection volume was set to 1 μl, the flow rate to 0.5 ml min^−1^ and column temperature to 30°C. Compounds were detected by the mass spectrometer operated in negative ion mode using multiple reaction monitoring (MRM). Ion spray voltage was set to −4.5 kV, gas 1 to 40 psi and gas 2 to 50 psi at a temperature of 500°C. Peaks were automatically detected based on retention time and MRM transition (Supplementary Table S1). Peak areas were normalized relative to the internal standards and concentrations were calculated according to the calibration curves using the software Analyst and MultiQuant (AB SCIEX, Framingham, MA, USA).

**Table 1:** Secondary metabolites and internal standards analyzed.

**Compound**	**Class**	**MW**	**Skeletal structure**	
*Trans*-piceatannol	Stilbene	244.24	1 (R = H)	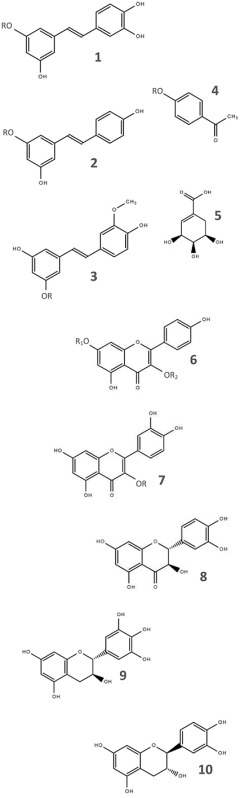
*Trans*-astringin	Stilbene	406.38	1 (R = glucose)	
*Trans-*resveratrol	Stilbene	228.24	2 (R = H)	
*Trans*-piceid	Stilbene	390.38	2 (R = glucose)	
*Trans*-isorhapontin	Stilbene	420.41	3 (R = glucose)	
Picein	Phenylpr.	298.29	4 (R = glucose)	
Shikimic acid	Cyclitol	174.15	5	
Kaempferol	Flavonoid	286.23	6 (R_1,2_ = H)	
Kaempferol 3-glucoside	Flavonoid	448.38	6 (R_2_ = glucose)	
Kaempferol 7-glucoside	Flavonoid	448.38	6 (R_1_ = glucose)	
Kaempferol 3-rutinoside	Flavonoid	594.52	6 (R_2_ = rutinose)	
Quercetin	Flavonoid	302.24	7 (R = H)	
Quercetin 3-glucoside	Flavonoid	464.38	7 (R = glucose)	
Taxifolin	Flavonoid	304.25	8	
Gallocatechin	Flavonoid	306.27	9	
Catechin	Flavonoid	290.27	10	
**INTERNAL STANDARDS**				
Naringin	Flavonoid	580.53		
Orientin	Flavonoid	448.38		
Pinosylvin	Stilbene	212.24		

*Compound name, compound class, molecular weight (MW), and skeletal structure are given*.

### Statistics

All values are given as mean ± standard error (SE). Differences were tested by the Student's *t*-test and correlation analyses were carried out using Pearson product-moment correlation after testing for normal distribution (Kolmogorov-Smirnov test) and equality of variances (Levene's test). All tests were conducted at a significance level of 5% using SPSS (IBM SPSS Statistics 21, IBM). Infection-induced concentration changes (in μmol g^−1^) were calculated as the positive or negative deviation of concentrations in infected needles from concentrations in healthy needles of the same tree and sampling date. Relative changes (in %) were calculated by dividing the absolute concentration change by the concentration in healthy needles.

## Results

### Variation in fungal infection intensity

The percent of infected current-year-needles during the last 5 years of the more susceptible trees ranged between 56 and 82%, whereas the tree with low susceptibility exhibited an infection intensity of 0.4% (Table 2). Variations between the analyzed years reflected the general trend in the region with highest infection pressure in 2011 (Fuchs et al., 2016).

**Table 2:** Infection degree of analyzed trees for the years 2011–2015 (percentage of infected needles in each needle age group) and mean infection degree.

**Tree**	**Site**	**Tree height (m)**	**Infection (%)**					
			**2011**	**2012**	**2013**	**2014**	**2015**	**Mean ± SE**
RIN-A	Rinn Kriegerkapelle	14.1	100	90	20	90	80	76.0 ± 12.8
RIN-B		13.1	30	90	30	50	80	56.0 ± 11.2
RIN-C		14.3	60	90	50	80	85	73.0 ± 6.9
RIN-D	Rinn Fluchtalweg	8.5	90	90	40	40	90	70.0 ± 11.0
RIN-E		10.2	90	80	90	50	90	80.0 ± 6.9
RIN-F		13.2	80	80	60	30	40	58.0 ± 9.1
PRA-A	Praxmar	10.3	90	80	60	90	90	82.0 ± 5.2
PRA-B		6.3	100	60	60	70	90	76.0 ± 7.3
PRA-C		9.8	100	40	40	70	90	68.0 ± 11.1
PRA-R	Praxmar	16.4	0	0	0	0	2	0.4 ± 0.4

### Seasonal accumulation patterns of phenolic compounds in healthy needles

**Figure 2:**
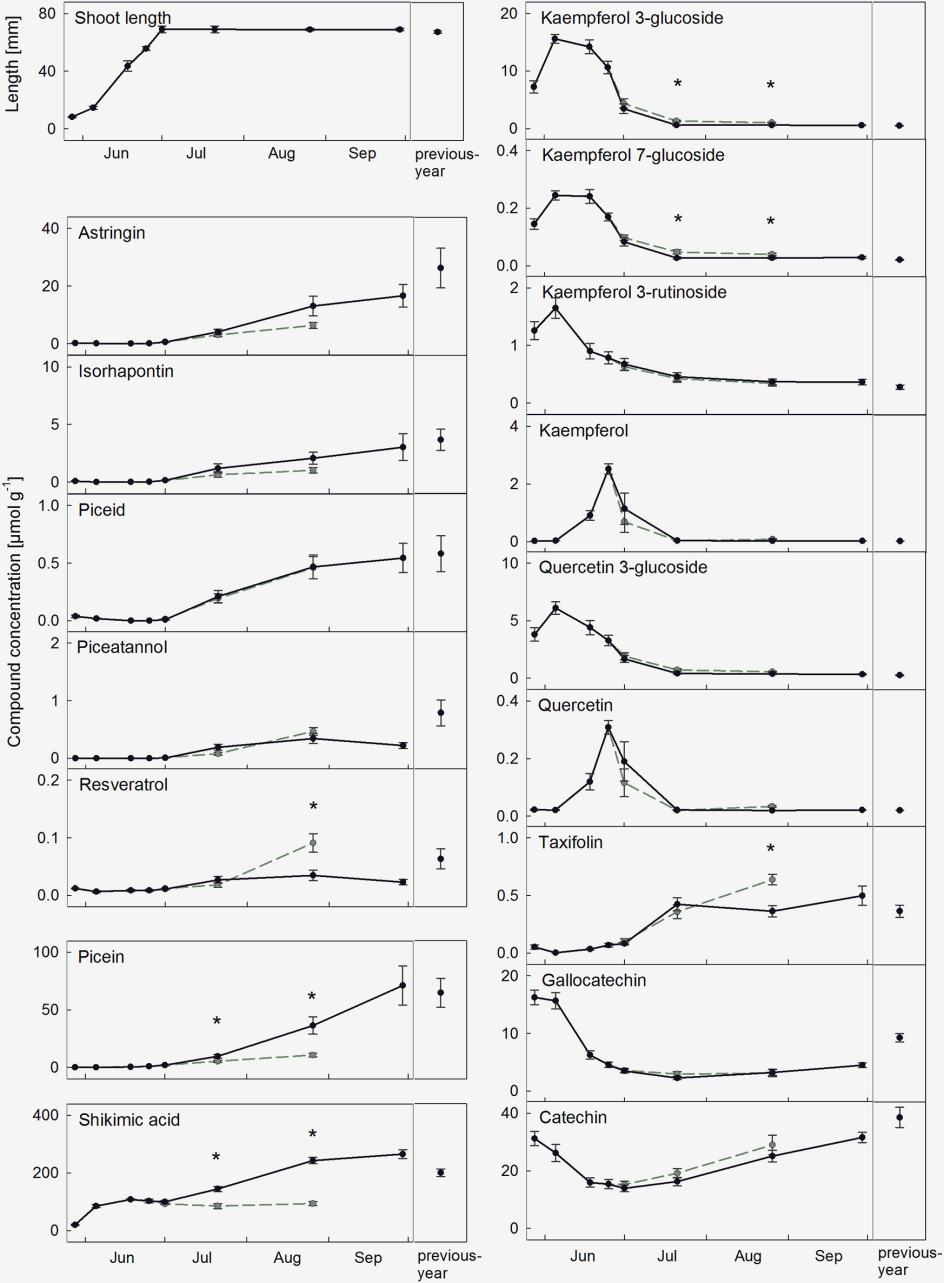
Accumulation of phenolic needle compounds during needle development in healthy needles (black symbols, mean ± SE, *n* = 9) and infected needles (gray symbols, mean ± SE, *n* = 9) from the end of May to the end of September, and in previous-year needles. All values are given as μmol g^−1^ dry weight, and significant differences between healthy and infected needles are marked with an asterisk. Accumulation patterns of the sum of total stilbenes and flavonoids and of the tree PRA-R are shown in Supplementary Figures S1, S2.

Current-year needles during development exhibited a significant increase of all stilbenes, starting about 4 weeks after bud swelling, and reaching compound-specific concentration levels at the end of summer (Figure 2 and Supplementary Figure S1). The concentration of picein showed a similar pattern, whereas shikimic acid had already accumulated in the unfolding buds, followed by a continuous increase. In contrast, flavonoids showed a more divergent pattern. Kaempferol, quercetin, and their glucosides showed distinct concentration peaks in the first weeks and then decreased rapidly. Glucosides appeared earlier, and decreased simultaneously together with the accumulation of their aglycones. The remaining flavonoids increased constantly from bud swelling (taxifolin) or showed more complex patterns with a decrease followed by an increase (catechin and gallocatechin). Overall, compound concentrations in previous-year needles corresponded to concentrations of current-year-needles at the end of September (Figure 2). Dominating compounds were shikimic acid (200.15 μmol g^−1^), followed by picein, (64.73 μmol g^−1^), the flavonoid catechin (38.59 μmol g^−1^), and the stilbene astringin (25.32 μmol g^−1^ dry weight).

### *Chrysomyxa* infection-induced effects on phenolics during progressive infection

First infection symptoms of attacked needles appeared at the beginning of July and most infected needles were shed by the end of September. During fungal infestation from July to August, a significant effect on several phenolic compounds was observed, with similar patterns within compound groups (Figure 2). Stilbene glucoside concentrations decreased slightly following infection (not significant), whereas the stilbene aglycone resveratrol increased up to 1.6-fold until August. Flavonoid glucosides increased up to 1.8-fold (partly significant), while the aglycones tended to decrease in the initial stage of infection. Picein and shikimic acid were significantly reduced by about 70 and 60%, respectively (Figure 2). Concentration changes of stilbenes, picein and shikimic acid in the needles augmented with proceeding fungal growth.

### Correlations of constitutive and induced phenolic levels with infection degree

The tree with distinctly lower susceptibility to *C. rhododendri* “PRA-R” showed a higher accumulation of several flavonoids and lower concentrations of gallocatechin, picein and shikimic acid at the beginning of July compared to the other trees (Table 3, Supplementary Figure S2). Especially kaempferol and taxifolin concentrations were 4.5- and 5.2-fold higher. Furthermore, following infection several stilbenes and flavonoid aglycones increased more and shikimic acid decreased less than in the more susceptible trees (Table 3). In contrast, flavonoid glucosides tended to increase less, especially at the initial phase of infection. Between July and August, induced concentration changes in PRA-R and in the more susceptible trees varied, but tended to drift even more apart (Table 3).

**Table 3:** Comparison of phenolic concentrations in the tree PRA-R with mean values of the more susceptible trees for constitutive levels at the beginning of July, and infection-induced concentration changes in mid-July and August.

	**Concentration beginning July (μmol g^−1^)**			**Inf.-ind. conc. changes mid-July (μmol g^−1^)**			**Inf.-ind. conc. changes August (μmol g^−1^)**		
	**Analyzed population**	**PRA-R**	**Correlation with inf. degree**	**Analyzed population**	**PRA-R**	**Correlation with inf. degree**	**Analyzed population**	**PRA-R**	**Correlation with inf. degree**
*Trans-*Astringin	0.50 ± 0.14	0.21	0.36	−1.02 ± 0.40	**+0.37**	−0.07	−6.72 ± 2.55	**+9.24**	**−0.88**
*Trans*-Isorhapontin	0.14 ± 0.04	0.07	0.32	−0.55 ± 0.18	−0.17	0.03	−1.05 ± 0.37	**+0.13**	**−0.77**
*Trans*-Piceid	0.01 ± 0.00	0.00	0.25	−0.02 ± 0.02	**+0.14**	−0.59	−0.01 ± 0.08	**+0.42**	**−0.79**
*Trans*-Piceatannol	0.00 ± 0.00	0.01	−0.36	−0.11 ± 0.04	**+0.68**	**−0.78**	+0.12 ± 0.06	**+0.62**	−0.43
*Trans*-Resveratrol	0.01 ± 0.00	0.01	−0.29	−0.01 ± 0.01	**+0.08**	**−0.85**	+0.06 ± 0.02	**+0.10**	−0.25
Kaempferol 3-glucoside	3.46 ± 0.85	**5.54**	−0.57	+0.70 ± 0.11	**+0.14**	0.25	+0.44 ± 0.06	**+0.29**	0.45
Kaempferol 7-glucoside	0.08 ± 0.01	0.10	−0.45	+0.02 ± 0.01	0.00	0.11	+0.01 ± 0.01	−0.00	0.41
Kaempferol 3-rutinoside	0.67 ± 0.10	**0.98**	−0.40	−0.03 ± 0.03	0.00	−0.36	−0.03 ± 0.02	**+0.04**	−0.19
Kaempferol	1.14 ± 0.55	**5.16**	**−0.66**	−0.00 ± 0.01	**+0.40**	**−0.94**	+0.06 ± 0.01	+0.07	−0.08
Quercetin 3-glucoside	1.67 ± 0.31	2.26	−0.41	+0.30 ± 0.08	**−0.01**	0.12	+0.18 ± 0.04	+0.09	0.24
Quercetin	0.19 ± 0.07	**0.99**	**−0.77**	−0.00 ± 0.00	**+0.06**	**−0.92**	+0.01 ± 0.00	+0.02	−0.19
Taxifolin	0.08 ± 0.01	**0.24**	**−0.88**	−0.07 ± 0.06	**+0.95**	**−0.76**	+0.27 ± 0.06	**+0.55**	0.00
Gallocatechin	3.49 ± 0.34	**1.94**	0.30	+0.67 ± 0.23	+0.76	0.35	−0.11 ± 0.30	**+2.61**	−0.18
Catechin	13.88 ± 1.14	15.01	−0.26	+2.88 ± 2.14	**+9.58**	−0.18	+3.88 ± 1.71	**+21.36**	−0.18
Picein	1.88 ± 0.44	**0.63**	0.14	−4.36 ± 1.19	−3.27	0.52	−25.63 ± 6.89	**−2.48**	**−0.82**
Shikimic acid	99.76 ± 4.00	**71.12**	0.45	−59.79 ± 4.77	**−45.49**	0.27	−149.57 ± 13.70	**10.99**	−0.59

*Infection-induced concentration changes (Inf.-ind. conc. changes) reflect the deviation of compound levels in infected needles from concentrations in healthy needles of the same tree and sampling date (for details see Section Materials and Methods). Also given are the Pearson-correlation-coefficients between compound concentrations and the mean infection degree including all trees (n = 10). Concentrations of the tree PRA-R outside the confidence interval of the analyzed population and significant correlations (p ≤ 0.05) are marked in bold*.

Correlation analyses between constitutive and induced phenolic compounds concentrations and the observed infection degrees including all analyzed trees revealed several significant negative relations (Table 3). Lower infection degrees were associated with higher constitutive and induced concentrations of kaempferol, quercetin, and taxifolin in July. Moreover, induced accumulation of stilbene aglycones in July and stilbene glucosides in August correlated negatively with the percentage of infected needles.

## Discussion

### Changes in phenolic profiles during needle development

The total amount of phenolic compounds was highest during sprouting and in autumn. Sprouting needles were characterized by high concentrations of flavonoids and their glucosides, mainly catechin, gallocatechin and kaempferol 3-glucoside. A few months old needles contained high concentrations of shikimic acid, picein, and stilbenes (the latter mainly represented by the glucosides astringin and isorhapontin; Figure 2). The good agreement of these results with published phenolic contents (Strack et al., 1989; Slimestad and Hostettmann, 1996; Slimestad, 1998) highlights universal accumulation patterns of phenolic compounds during needle development in Norway spruce, independent of study site and elevation. However, concentration levels can be influenced by genetic and environmental factors. For example, a positive correlation between concentration and elevation was reported for picein (Bahnweg et al., 2000). Furthermore, variations between different provenances and significant genetic regulation was reported for several stilbenes and flavonoids (Slimestad, 1998; Evensen et al., 2000; Lieutier et al., 2003; Ganthaler et al., 2017). The pronounced high turnover of the flavonoids quercetin and kaempferol in sprouting needles together with successive occurrence of glucosides and their aglycones (Figure 2) indicates a rapid modification of these compounds by de-glycosylation. However, the several times lower concentrations measured for aglycones compared to glycosides suggest that these compounds undergo further modification. Strack et al. (1989) supposed that kaempferol 3-glucoside is polymerized and translocated to the cell wall within the first weeks after sprouting.

### Infection-induced phenolic responses

Concentration changes of individual phenolic metabolites after infection can vary considerably among different host-pathogen systems, due to the complex interactions and functions of the compounds. Infection by *C. rhododendri* caused an increase in several flavonoids and resveratrol and a reduction of picein and shikimic acid in spruce needles (Figure 2). Similarly, in spruce infected by other fungal pathogens an increase of taxifolin (Evensen et al., 2000; Krajnc et al., 2014) and kaempferol 3-glucoside (Bahnweg et al., 2000) was observed. By contrast, shikimic acid is an important precursor for the synthesis of phenylalanine and subsequently for the main flavonoid synthetic pathway (Vogt, 2010), and may decline due to enhanced flavonoid production. Except for taxifolin, the concentrations of the mentioned compounds changed significantly both at the initial and late stages of infection, but with varying extent (Figure 2). Significant infection stage-dependent changes in phenolics were also found in phloem tissue infected by *Endoconidiophora polonica* (Krajnc et al., 2014) and willow infected by *Melampsora* rust (Hakulinen et al., 1999). This may be due to the complex infection-triggered signal transduction and activation of metabolic pathways, with a primary accumulation of monomers, which then are gradually modified and converted into insoluble products to isolate the fungus (Matern and Kneusel, 1988). Furthermore, the temporal variability of phenolic concentrations may challenge the fungus by making the conditions even more unpredictable, as pointed out by Edenius et al. (2012) for leaf-eating invertebrates in spruce forests. The observed decrease in concentration of some compounds may be related to plant response, but also to metabolization by the fungus, as reported for stilbenes and *E. polonica* (Hammerbacher et al., 2013).

### Relationship between phenolics levels and susceptibility

Both constitutive levels of several compounds at the beginning of July and infection-induced concentration changes were correlated with the infection degree of the trees (Table 3). Interestingly, constitutive and early stage infection-induced concentrations of the flavonoids kaempferol, quercetin, and taxifolin strongly correlated with susceptibility and also showed remarkable concentration levels in the resistant tree PRA-R. Notably, taxifolin concentration increased ~30-fold in infected needles relative to healthy needles in PRA-R compared to a 1.7-fold rise in the more susceptible trees. These aglycones may be effective against the pathogen when incorporated into the plant cell wall, thereby preventing nutrient uptake by haustoria in the initial infection phase (Matern and Kneusel, 1988; Fossdal et al., 2012). Furthermore, the infection-induced accumulation of stilbenes seems to be related to better defense of the tree. In the early infection phase, the accumulation of the aglycones piceatannol and resveratrol was correlated with lower susceptibility, whereas in the later infection phase in August, this applied to the glucosides astringin, isorhapontin, and piceid, likely because of proceeding glycosylation for higher compounds stability (Levin, 1971; Slimestad and Hostettmann, 1996). The antifungal effect of stilbenes was reported in several studies (Chong et al., 2009; Hammerbacher et al., 2011; Plumed-Ferrer et al., 2013) and may be one reason why older needles with a high constitutive stilbene level are never infected by *C. rhododendri*. A strong correlation between isorhapontin concentrations and susceptibility was also found in spruce phloem infected by *E. polonica* (Brignolas et al., 1998), and astringin was shown to limit the depth of hyphal penetration by *Heterobasidion annosum* in the bark (Lindberg et al., 1992). The direct toxicity of hydroxystilbenes can be related to their capacity to disrupt cell membranes, nuclear and mitochondrial membranes in fungal germ tubes (Pezet and Pont, 1990).

The tree PRA-R represents an extreme example within the range of susceptibility analyzed in this study. The data for this tree, if considered on its own, allow only limited interpretation. Nonetheless, significantly higher constitutive and induced concentrations of several phenolic compounds compared to the more susceptible trees (Table 3, Supplementary Figure S2) underline the detected correlations with infection degree within the entire population. Moreover, results of PRA-R may help to identify characteristic defense strategies of spruce to needle rust, which should be part of future analyses, preferably including more trees with enhanced resistance.

Our results indicate that for the development of enhanced resistance to *C. rhododendri* a fast and strong infection-induced response of the tree may be even more important than constitutively highly expressed phenolic levels. This finding is in agreement with data regarding tree resistance to *H. annosum*, which was correlated with a capability to induce phenolic defense compounds (Brignolas et al., 1995; Danielsson et al., 2011; Schiebe et al., 2012). In addition, fast infection-induced activation of the phenylpropanoid and downstream pathways was repeatedly found in trees with enhanced resistance (Danielsson et al., 2011; Hammerbacher et al., 2011). However, pathogen defense may also depend on a capability to rapidly modify preformed compounds. When challenged with the pathogen, the tree with low susceptibility exhibited smaller increases in flavonoid glucosides, but produced significantly more of their aglycones than the more susceptible trees (Table 3), indicating higher enzymatic activity of glucosidases and enhanced de-glycosylation. Aglycones are considered to have greater antifungal activity (Levin, 1971), and consequently, rapid formation of aglycones in response to injury or fungal infection is considered an important defense strategy (Nicholson and Hammerschmidt, 1992).

## Conclusion

The phenolics profile of Norway spruce needles changed significantly during needle development, an important aspect to be considered in the timing of sampling for host-pathogen interaction studies. Infection-induced responses of the trees included changes in the concentrations of stilbenes, flavonoids, picein and shikimic acid and influenced the infection intensity. Especially a fast accumulation of flavonoid aglycones was associated with fungal growth and formation of infection symptoms. Thus, it is likely that lower susceptibility of individual trees is based on a combination of enhanced constitutive and inducible phenolic defense mechanisms, which appears to be a common strategy of Norway spruce (see Lieutier et al., 1997, 2003; Fossdal et al., 2012). Our results improve the physiological and biochemical understanding of pathogen defense in Norway spruce and a systematic screening for high concentrations of key phenolic metabolites could enable rapid identification of plant material that is resistant to *C. rhododendri*. Thus, the data may facilitate the selection of promising trees for breeding programs, as has been proposed as a useful strategy for combating a variety of pests and pathogens (Leather, 1996; Witzell and Martín, 2008; Herrmann and Schauer, 2013).

## Author contributions

Conceived and designed the experiments, contributed to the writing and revision of the manuscript: SM, AG, WS, and IK. Performed the experiments and analyzed the results: AG and WS.

### Conflict of interest statement

The authors declare that the research was conducted in the absence of any commercial or financial relationships that could be construed as a potential conflict of interest.
