# Supplementary material for: Foliar Phenolic Compounds in Norway Spruce with Varying Susceptibility to Chrysomyxa rhododendri: Analyses of Seasonal and Infection-Induced Accumulation Patterns
Source: Front Plant Sci. 2017 Jun 30;8:1173. doi: 10.3389/fpls.2017.01173 (PMC5492020; doi:10.3389/fpls.2017.01173)
Supplement: Supplementary file 1 [file DataSheet1.pdf]

Supplementary material

Foliar phenolic compounds in Norway spruce with varying susceptibility to *Chrysomyxa rhododendri*: analyses of seasonal and infection-induced accumulation patterns

Andrea Ganthaler, Wolfgang Stöggl, Ilse Kranner, Stefan Mayr

Supplementary Table S1. MRM transitions, settings and retention time (rt) for analyzed compounds and internal standards.

Q1 Mass (Da)	Q3 Mass (Da)	Time (msec)	ID	DP (volts)	CE (volts)	CXP (volts)	rt	Internal standard
405.0	243.0	10	astringin	-120	-26	-13	1.75	orientin
405.0	159.0	2	astringin	-120	-60	-7		orientin
288.9	245.0	10	catechin	-85	-22	-13	1.45	orientin
288.9	203.0	2	catechin	-85	-26	-15		orientin
419.1	257.1	10	isorhapontin	-95	-22	-9	2.10	naringin
419.1	241.1	2	isorhapontin	-95	-46	-9		naringin
447.0	284.0	10	kaempferol 3-glucoside	-110	-38	-15	2.22	naringin
447.0	255.0	2	kaempferol 3-glucoside	-110	-54	-17		naringin
446.9	284.9	10	kaempferol 7-glucoside	-130	-34	-11	2.25	naringin
446.9	151.0	2	kaempferol 7-glucoside	-130	-46	-7		naringin
593.0	284.9	10	kaempferol 3-rutinoside	-145	-42	-11	2.13	naringin
593.0	254.9	2	kaempferol 3-rutinoside	-145	-68	-11		naringin
343.0	135.1	10	picein-formate	-60	-16	-7	1.19	orientin
343.0	44.9	2	picein-formate	-60	-54	-5		orientin
284.9	185.0	10	kaempferol	-110	-36	-9	3.19	pinosylvin
284.9	187.0	2	kaempferol	-110	-40	-9		pinosylvin
300.9	150.9	10	quercetin	-95	-28	-11	2.81	naringin
300.9	178.9	2	quercetin	-95	-26	-7		naringin
463.0	300.0	10	quercetin 3-glucoside	-125	-38	-15	2.06	naringin
463.0	301.0	2	quercetin 3-glucoside	-125	-32	-15		naringin
304.9	124.9	10	gallocatechin	-55	-24	-9	1.06	orientin
304.9	179.0	2	gallocatechin	-55	-24	-3		orientin
242.9	159.0	10	piceatannol	-105	-36	-11	2.25	naringin
242.9	201.0	2	piceatannol	-105	-28	-13		naringin

389.0	227.0	10	piceid	-95	-26	-9	2.02	naringin
435.0	227.0	10	piceid-formate	-40	-30	-9		naringin
435.0	389.2	2	piceid-formate	-40	-14	-9		naringin
302.9	285.0	10	taxifolin	-80	-16	-5	2.14	naringin
302.9	125.0	2	taxifolin	-80	-26	-9		naringin
172.9	92.9	10	shikimic acid	-60	-22	-7	0.37	orientin
172.9	111.0	2	shikimic acid	-60	-14	-9		orientin
226.9	185.0	10	resveratrol	-90	-26	-9	2.61	naringin
226.9	143.0	2	resveratrol	-90	-34	-9		naringin
579.1	271.0	10	naringin	-140	-44	-11	2.18	/
579.1	459.2	2	naringin	-140	-36	-9		/
211.0	169.0	10	pinosylvin	-90	-26	-5	3.52	/
211.0	167.1	2	pinosylvin	-90	-24	-7		/
447.0	327.0	10	orientin	-120	-30	-15	1.84	/
447.0	357.0	2	orientin	-120	-30	-19		/

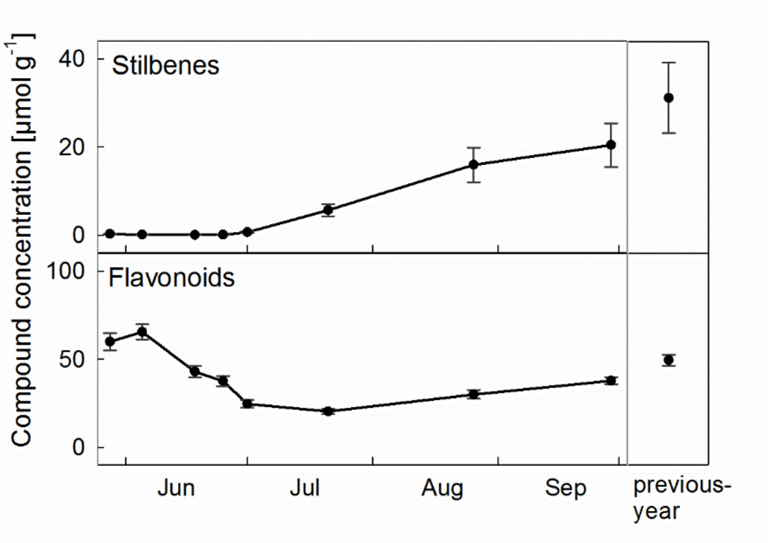

Supplementary Figure S1. Accumulation of total stilbenes and flavonoids during needle development in healthy needles (mean ± SE, n = 9) from end of May to end of September and in previous-year needles. All values given as µmol g^{-1} dry weights.

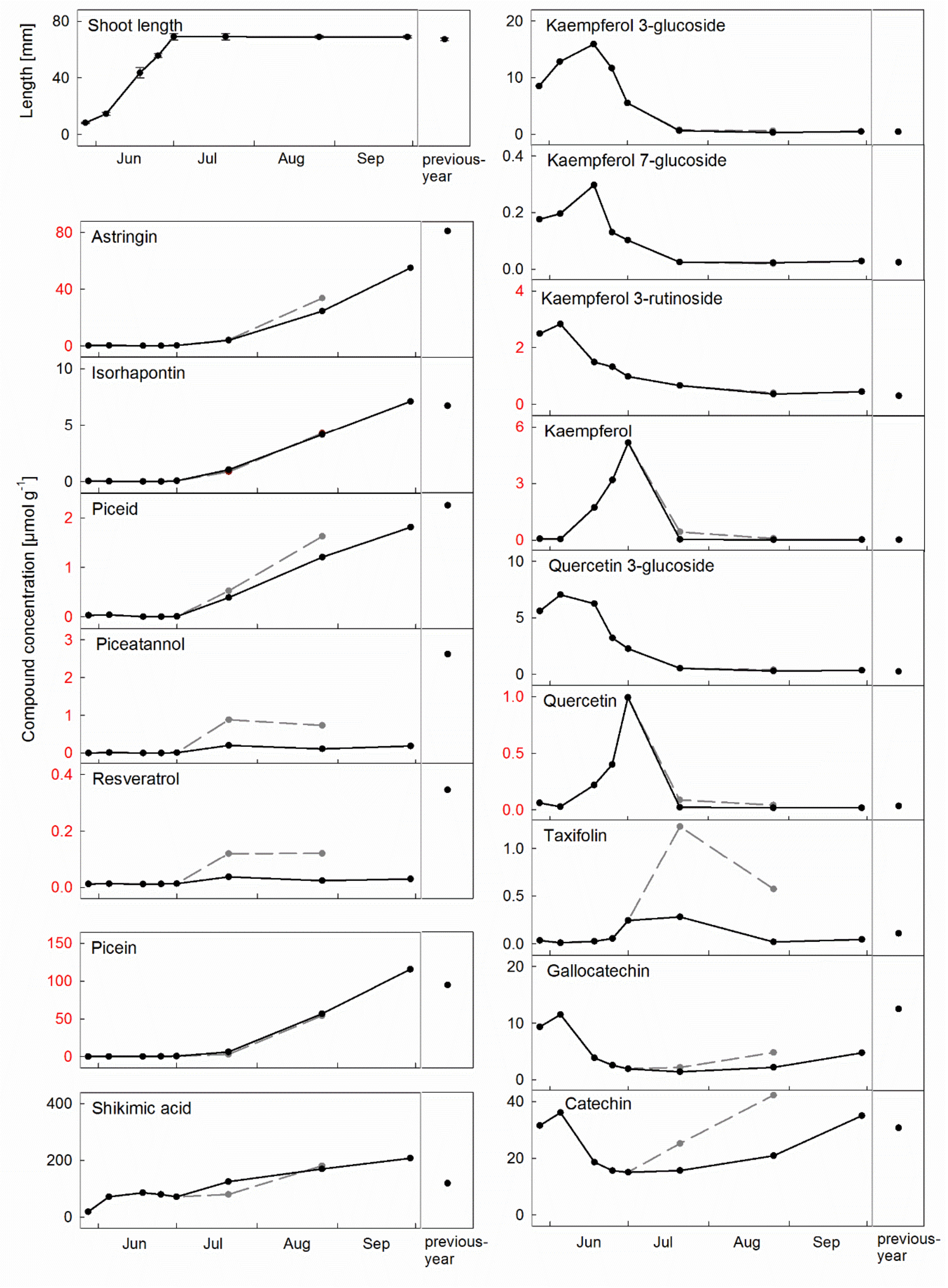

Supplementary Figure S2. Accumulation of phenolic needle compounds during needle development in healthy needles (black symbols) and infected needles (grey symbols) of the tree with low susceptibility PRA-R. All values are given as µmol g^{-1} dry weight. Please note that the scaling of several y-axes (shown in red) does not conform to the scaling in Figure 2 due to distinct higher concentrations of several compounds in the tree PRA-R.
